# Associations between Local and State Structural Stigma, Minority stress, and Mental Health in a Nationwide Sample of Sexual Minority Adolescents

**DOI:** 10.1007/s10560-025-01068-0

**Published:** 2025-12-28

**Authors:** Rory P. O’Brien, John R. Blosnich, Julie A. Cederbaum, Laura Ferguson, Michael Hurlburt, Dorian E. Traube, Luis A. Parra, Harmony Rhoades, Sheree M. Schrager, Jeremy T. Goldbach

**Affiliations:** 1 Suzanne Dworak-Peck School of Social Work, University of Southern California, 669 W 34th St, Los Angeles, CA 90089 USA; 2 San Diego State University School of Social Work, 5500 Campanile Drive, San Diego, CA 92182 USA; 3 Institute on Inequalities in Global Health, University of Southern California, 1845 N. Soto St, Los Angeles, CA 90032 USA; 4 George Warren Brown School of Social Work, Washington University in St Louis, One Brookings Dr, St Louis, MO 63130 USA; 5 Department of Psychology, University of California, Davis, 135 Young Hall, One Shields Avenue, Davis, CA 95616 USA; 6 Graduate Studies & Research, California State University, Dominguez Hills 1000 E. Victoria St, Carson, CA 90747 USA

**Keywords:** Structural stigma, Policy, LGBTQ adolescents, Minority stress, Mental health

## Abstract

Structural stigma, which includes policies and attitudes that mark minorities for discrimination, contributes to sexual minority adolescents’ (SMA) stress and mental health disparities, yet the relative contributions of county- vs. state-level stigma remain unknown. This study examined how structural stigma affects SMA exposure to minority stress and mental health symptoms with a U.S. national sample of SMA (*N* = 2,558, ages 14-17, *M* = 15.9), of whom 64.35% were cisgender designated female at birth and 48.28% were bisexual/pansexual. Researchers analyzed links between county- and state-level stigma with anxiety and depression symptoms and assessed whether minority stress mediated these associations. Minority stress significantly linked structural stigma to mental health symptoms. Adolescents in supportive counties and supportive states experienced fewer minority stressors and mental health symptoms compared to those in unsupportive counties of unsupportive states. Locally supportive counties and local action to address structural stigma may protect SMA mental health, even in otherwise unsupportive state contexts.

Sexual minority adolescents (SMA; i.e., adolescents who identify as lesbian, gay, bisexual, pansexual, queer, or who have non-heterosexual sexual identities) are more likely than their heterosexual peers to report significant mental health concerns (Kann et al., 2018). In the minority stress framework, heightened mental health symptoms are largely attributed to chronic, negative experiences, including both distal (e.g., enacted discrimination) and proximal (e.g., internalized homonegativity) stress exposures stemming from stigmatization (Meyer, 2003). Among SMA, the unique stressors related to stigmatized social status are associated with increased anxious (D’augelli, 2002; Parra et al., 2018) and depressive (Baams et al., 2018; Hall, 2018; Williams et al., 2005) symptoms, substance use (Goldbach et al., 2014), and suicide attempt (Fulginiti et al., 2020; Saewyc et al., 2020). Minority stress-mental health associations during adolescence can also negatively affect SMA’s psychosocial adjustment and development. For SMA, minority stressor exposure and mental health problems are interrelated with low self-esteem, devaluation of the self, and challenges with developing and affirming their sexual minority identities and behaviors, as well as having fewer coping resources to contend with minority stressor exposure (Russell & Fish, 2016, 2019). These SMA-specific developmental processes in the context of minority stressor exposure and mental health are highly salient during adolescence, constituting a developmental period associated with greater self-awareness to peer acceptance or rejection (Dahl & Gunnar, 2009), the emergence of sexual attractions and sexual identity exploration and formation (Hall et al., 2021; Patterson, 2008), and greater risk for severe and pervasive mental health problems that could persist over the lifetime (De Girolamo et al., 2012; Kessler et al., 2007).

Prejudice embedded in, encouraged, or permitted by social structures can shape stress exposure and drive health disparities (Hatzenbuehler & Link, 2014), commonly referred to as *structural stigma*. For example, same-sex marriage laws and neighborhood hate crime incidence rates have each been linked to patterns in SMA mental health (Duncan et al., 2014; Raifman et al., 2017). Likewise, proposed state legislation antagonistic toward sexual minority rights is linked to increased sexual minority stress and past-year suicide attempt (O’Brien et al ., 2023). Though typically operationalized in terms of policies and laws, structural stigma is measured in a variety of ways, including voting patterns, sociopolitical opinions, and social service access indices to explore the multiple structures that pattern social and behavioral health disparities (Hatzenbuehler, 2018). Indeed, existing research suggests that structural stigma has a clear relationship with individual level mental health (Chien et al., 2022; Hatzenbuehler, 2018; Perez-Brumer et al., 2017; Rabasco & Andover, 2020; Saewyc et al., 2020); O’Brien et al ., 2023).

Despite progress, gaps remain in our understanding of structural stigma and individual level mental health. One critical gap addressed in the current work is that studies on structural stigma have generally focused on associations between individual health and structural stigma at a single societal level, such as the state or school district level. For example, Hatzenbuehler and Keyes (2013) examined associations between the proportion of school districts that have anti-bullying policies (a county-level analysis) and student self-reported past year suicide attempt. Another gap central to this study is that the limited research which has considered structural stigma at local and macro levels has been descriptive. Cramer et al. (Cramer et al., 2017), identified a greater amount and topical variety of legislation on sexual orientation at the state than county level. Despite calls for further research (Cramer et al., 2017), few studies have explored structural stigma and SMA health at the local level, such as the municipality or county level (Duncan et al., 2014; Hatzenbuehler & Keyes, 2013; Saewyc et al., 2020). One recent study identified that sexuality-based experiences of discrimination and state-level structural stigma each uniquely contribute to SMA mental health problems, though structural stigma was no longer significant when adjusting for stressors such as proximal racist, xenophobic, and weight discrimination (Gordon et al., 2024). Another study, on structural sexism, included macro-, meso-, and micro-level factors in one random effects model (Homan, 2019), with results indicating that each level independently affects women’s self-reported health, chronic health, and physical functioning. The disentanglement of the effects of stigma at multiple levels of society on SMA health is yet to be explored and may improve understanding of how stigma affects health and well-being.

Although minority stress theory (Meyer, 2003) and social stress theory (Meyer et al., 2008) each posit that structural stigma’s association with mental health can be explained through individual-level minority stress experiences, studies have rarely explicitly tested this assertion, generally due to use of limited psychosocial measures or sample sizes too small to detect effects (Hatzenbuehler, 2018; Pachankis et al., 2014). Thus, the current study examined associations between both county- and state-level structural stigma and individual level minority stress experiences and, in turn, anxious and depressive symptoms among SMA, utilizing vetted measures of psychosocial functioning and a large-scale sample of SMA. First, we hypothesized that residence in counties and/or states with more protections will be associated with fewer experiences of recent (past 30-day) minority stress, anxious symptoms, and depressive symptoms. Specifically, minority stress and anxious and depressive symptoms were expected to be lower among SMA living in supportive counties compared to unsupportive counties, and lowest in supportive states. Second, we hypothesized that the effects of structural stigma on both anxiety and depression symptoms would be indirectly associated through minority stress.

## Materials & Methods

### Description of Data

Data are from a nationwide sample of SMA ages 14–17 (*N* = 2,558) enrolled in the Adolescent Experiences over Time Study (NIH R01MD012252); details on the study protocol have been published previously (Schrager er al., 2022). Participants completed a baseline online survey between late 2018 and early 2019 on their experiences of minority stress and mental health symptoms. Participants were recruited via social media advertisements on Facebook, Instagram, and YouTube or via respondent-driven sampling where eligible participants received additional compensation ($10 per referral, up to three total) for referring additional eligible participants. The study obtained institutional review board approval and a waiver of parental consent from the University of Southern California; all participants provided informed assent prior to completing the study.

Participants were eligible if they were 14–17 years old, lived in the United States, were not “100% heterosexual” per *Add Health* guidelines (Harris et al., 2009), and identified as cisgender at baseline. All participants had to identify as cisgender SMA at study baseline because the study’s primary aim was to understand sexual minority stress longitudinally using the Sexual Minority Adolescent Stress Inventory (SMASI; Schrager er al., 2022). Although subsequent research with the SMASI has established its validity with trans and nonbinary SMA (Fulginiti et al., 2020), the SMASI had not yet been validated with trans and nonbinary SMA at baseline recruitment into this study.

## Measures

### County and State Structural Stigma

We used respondent zip codes to code adolescents into states and counties. SMA who reported zip codes that included multiple counties were designated to the county that contained the greater population proportion of the zip code (*n* = 742). For most of this subsample of adolescents (*n* = 572), the counties that participants’ zip codes crossed into all had the same county-level structural stigma score (see county structural stigma scoring below), such that those adolescents are still reliably grouped into the correct county stigma group. A sensitivity analysis was conducted excluding the remaining 170 adolescents.

To operationalize state-level stigma, adolescents were coded on a scale of 1–4 corresponding to the Human Rights Campaign (HRC) State Equality Index (Warbelow et al., 2019) ratings for their state of residence in the year they completed the survey (2018 or 2019). The HRC monitors state laws and then assigns states to four groups (what HRC calls “High priority to achieve basic equality,” “Building equality,” “Solidifying equality,” and “Working toward innovative equality,” to indicate their policy targets), interpreted here as 1=*most unprotective*, 2=*unprotective*, 3=*protective*, and 4=*most protective*. These designations are based on a count of each states’ policies (from a list of more than 55 policies) that affect LGBTQ people, including laws on parenting, hate crimes, adolescents protections, and non-discrimination. Fourteen of the policies specifically concerned LGBTQ adolescents (such as anti-bullying policy mandates for schools); however, most of the state policies in the index can plausibly affect adolescents wellbeing. Only a few states fell into groups 2 (e.g., Utah and Wisconsin) and 3 (e.g., Hawaii and Iowa), so the score was dichotomized with 1–2 representing less protective and 3–4 representing more protective states.

Three county stigma indicators were also created for this study. County contexts were coded based on the county-level results of the 2016 presidential election (1=Clinton, 0=Trump; (Guardian, 2016) and the presence/absence (1=*Yes*, 0=*No*) of county or city non-discrimination ordinances (*Local Nondiscrimination Ordinances*) and conversion therapy bans (assessed via review of city and county measures, agendas, and municipal codes). Counties in states with statewide non-discrimination ordinances and/or conversion therapy bans were coded as having these protections at the county level, following similar conventions in the literature (Cramer et al., 2017).

The sum of the binary county stigma indicators was recoded into a categorical variable representing the protectiveness of each county: low (0 protective indicators, *n* = 688), medium (1–2 protective indicators, *n* = 849), and high (all 3 protective indicators, *n* = 1,020), allowing comparison of counties with none, some, and all indicators.

Finally, the state and the county structural stigma scores were combined into a single 4-group variable of adolescents living in unprotective counties in unprotective states (low-low, *n* = 688, hereafter, “LL”), medium or highly protective counties in unprotective states (med-low, *n* = 647, “ML”), medium protective counties in highly protective states (med-high, *n* = 311, “MH”), and highly protective counties in highly protective states (high-high, *n*=911, “HH”). The ML group includes both medium or highly protective counties in unprotective states because there were few adolescents living in highly protective counties in unprotective states and a preliminary ANOVA analysis indicated that these groups’ mean minority stress score did not significantly differ. No adolescents resided in highly unsupportive counties (county=0) of supportive states (state=1), as state non-discrimination and/or conversion therapy laws were often imposed by states upon counties. The decision to combine county and state variables, rather than test an interaction, was due to these low and empty cell sizes for the high county-low state and low county-high state groups. While a multilevel approach, which would not involve computing this combined county-state variable, was also considered, it was not deemed necessary given that variance in anxious and depressive symptoms was not notably attributable to adolescents’ county or state of residence (all intraclass correlations were less than 1%).

### Adolescent Minority Stress

Participants completed the 54-item SMASI Schrager er al., 2022, Goldbach et al ., 2021 to assess experiences of minority stress. SMA were asked if they experienced each item in the past 30 days, such as “*I have felt unsafe or threatened in the neighborhood where I live because I am LGBTQ”* (0 = *No*, 1 = *Yes*). Higher summed scores indicated recent exposure to more minority stressors. The measure had high internal consistency (ω=0.91), assessed using the ordinal omega coefficient to account for binary response options.

### Anxiety Symptoms

The Generalized Anxiety Disorder 7-item questionnaire (GAD-7; (Spitzer et al., 2006) asked participants to rate anxiety-related symptoms in the past 2 weeks. Participants rated items (e.g., “*feeling nervous*,* anxious*,* or on edge*,” “*trouble relaxing*”) on a 4-point Likert scale (0=*Not at all* to 3=*Nearly every day)*. Higher summed scores suggested elevated anxiety symptoms. The GAD-7 had high internal consistency with a Cronbach α=0.90.

### Depression Symptoms

The Center for Epidemiological Studies Depression Scale (CES-D 4-item) (Melchior et al., 1993) asked participants to rate the frequency of depression symptoms (e.g., “*I felt depressed*,” “*I had crying spells*”) in the past 7 days. Items were scored on a 4-point scale from 0 = “*Rarely or none of the time*” to 3 = “*Most or all of the time.*” The CES-D4 had good internal consistency with a Cronbach α=0.84.

### Covariates

Adolescents were asked to describe their sexual orientation. Qualitative responses were coded into major identity groups, including Gay or Lesbian, Bisexual, Pansexual, Queer, and Another sexual orientation. Analysis of variance (ANOVA) indicated a lack of significant mean differences between the bisexual, pansexual, queer, and “another” groups in minority stress, anxious, and depressive symptoms, so we recoded participants as 0 = *Bi/Pan/Queer/Another* and 1 = *Gay/Lesbian* variable for parsimony in path analysis. Adolescents also self-identified whether they were 0 = *designated female at birth* or 1 = *designated male at* birth.

Adolescents self-identified their race/ethnicity from a list of options, including White/Caucasian, Black/African American, Latinx/Hispanic, Asian/Pacific Islander, Native American/American Indian/Alaskan Native, and Multiracial. Research shows that SMA people of color experience more stressors at the intersections of their race or ethnicity (Cyrus, 2017). Thus, we ran ANOVAs that indicated that groups of people of color did not generally differ from each other in minority stress or outcomes, so we then recoded the variable as 0 = *People of Color* and 1 = *White* for parsimony in path analysis.

Adolescents reported whether they were eligible to receive free- or reduced-price lunch at school, which was used as a proxy for low socioeconomic status (0=*No*, 1=*Yes;* (Domina et al., 2018); respondents who replied “I don’t know” were recoded into the “No” referent group. Participants’ zip codes were used to assign a binary indicator of urbanicity (0=*rural*, 1=*urban*) based on the zip code’s Rural-Urban Commuting Area version 3.1 (RUCA; (Center, 2022) score. Rural zip codes include micropolitan areas, low commuter areas, and small towns, whereas urban zip codes include adolescents in high density metropolitan areas.

## Data Analysis

To test the study hypothesis, we constructed a path analysis testing whether effects of county-state structural stigma on anxiety and depression symptoms were indirectly linked through minority stress using the lavaan package version 0.6–5.6 (Rosseel, 2012) in R 4.1.2. The model produced direct effects for each county-state structural stigma group on the mental health outcomes (anxiety symptoms regressed on ML (c’_*1*_), MH (c’_*2*_), and HH (c’_*3*_) and depression symptoms regressed on the same groups (c’_*4*_, c’_*5*_, and c’_*6*_), all with the LL group as referent). Indirect effects were computed by multiplying paths between each county-state structural stigma group and recent minority stress (*a*_*1*_, *a*_*2*_, and *a*_*3*_, with the LL group as referent) with the effects of recent minority stress on anxiety symptoms (*b*_*1*_) and depression symptoms (*b*_*2*_). Indirect associations met mediation criteria if 95% BCa CIs did not include zero, regardless of the significance of direct and total effects (MacKinnon, 2012; Preacher & Hayes, 2008; Zhao et al., 2010).

We obtained BCa CIs using bootstrapping with 10,000 iterations to account for the non-normality of variables and indirect effects (Montoya & Hayes, 2017). We assessed model fit with the comparative fit index (CFI), Tucker-Lewis index (TLI), normed-fit index (NFI), root mean square error of approximation (RMSEA), and standardized root mean square residual (SRMR). CFI, TLI, and NFI thresholds of >0.95 and RMSEA and SRMR thresholds of <0.06 and <0.08, respectively, indicated a good fit of the data to the model. We covaried predictors (including county-state groups) with each other, such that estimates represent associations between residual variances not accounted for by other predictors. We likewise covaried anxiety and depression symptoms in the model to account for their correlation (*r* =.62, *p* <.05). Missingness was minimal (*n*=18) and statistical models were estimated with full information maximum likelihood.

**Table 1:** Demographic, policy characteristics, and mental health (*n* = 2,558)

	%/M (*n*/SD)
*Age* (range: 14–17)	15.90 (0.98)
*Race and ethnicity*	
White	60.36 (1,544)
Latino/Hispanic	14.43 (369)
Multiracial/multiethnic	9.54 (244)
Black οr African American	7.74 (198)
Asian/Pacific Islander	6.18 (158)
American Indian/Alaska Native	1.13 (29)
Another race/ethnicity	0.63 (16)
*Sex Designated at Birth*	
Female	64.35 (1,646)
Male	35.65 (912)
*Sexual orientation*	
Gay/Lesbian	43.12 (1,103)
Bisexual/Pansexual	48.28 (1,235)
Queer	2.66 (68)
Another sexual orientation (questioning, asexual, etc.)	5.94 (152)
*Eligible for free/reduced price lunch at school*	
Yes	39.25 (1,004)
No	60.09 (1,537)
*Region*	
West	24.63 (630)
Southwest	13.14 (336)
Midwest	17.51 (448)
Southeast	23.14 (592)
Northeast	21.58 (552)
*Urbanicity*	
Urban	80.30 (2,054)
Rural	19.70 (504)
*County - State Structural Stigma*	
Unsupportive County in Unsupportive State (Low-Low)	26.94 (689)
Medium Supportive County in Unsupportive State (Med-Low)	25.41 (650)
Medium Supportive County in Supportive State (Med-High)	12.12 (310)
Supportive County in Supportive State (High-High)	35.54 (909)
*Recent Minority Stress* (SMASI^1^, range: 0–54)	13.23 (8.74)
*Anxiety Symptoms* (GAD-7, range: 0–21)	11.80 (5.96)
*Depression Symptoms* (CES-D4, range: 0–12)	6.32 (3.41)

^1^The Sexual Minority Adolescent Stress Inventory

## Results

The majority of the sample were designated female at birth (64.4%), White (60.4%), living in urban environments (80.3%), and ineligible for free- or reduced-price lunch (60.1%). Nearly half identified as bisexual and pansexual (48.3%), and respondents represented all regions of the United States except for U.S. territories. See Table 1 for study demographics.

**Fig. 1:**
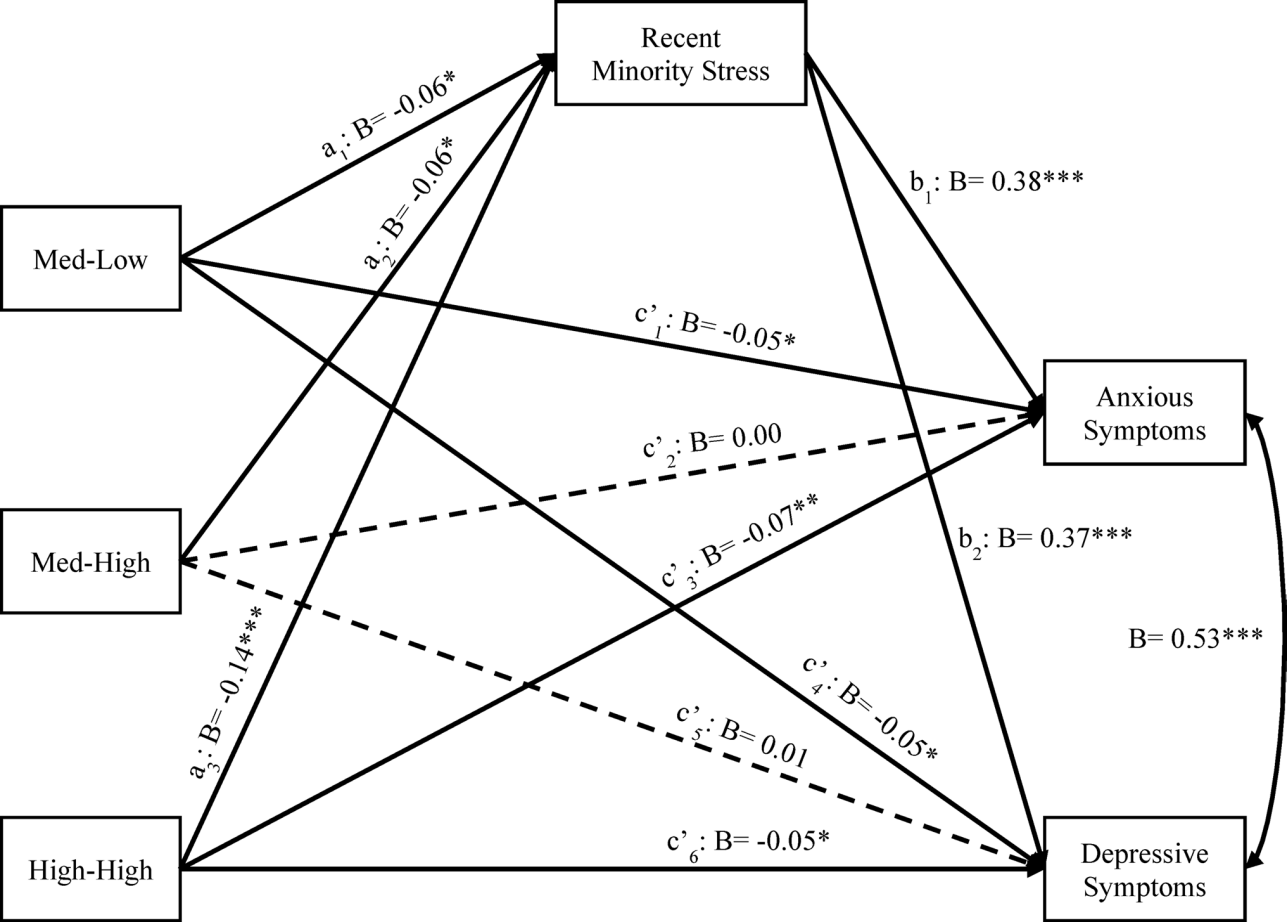
A conceptual and statistical diagram of the model’s predictors, mediator, and outcomes variables. *Notes*: Solid arrows indicate statistical significance, *p<.05, **p<.01, and ***p<.001. Betas (**B**) are standardized regression estimates. Indirect and total effects are detailed in Table 2. Covariates and covariances are presented in Table 3 and not included here for parsimony. This figure was created using Microsoft PowerPoint.

We used path analysis to test whether minority stress indirectly linked associations among county-state groups and anxiety and depression symptoms. Complete results are reported in Tables 2 and 3 and main findings with standardized coefficients to aid interpretation are illustrated in Figure 1. Based on bivariate tests (see Supplemental Table 1), covariates not associated with outcomes were excluded from the path analysis, with improvements in model fit. The data had good fit to the model (CFI = 0.999, TLI = 0.967, NFI = 0.999, RMSEA = 0.033, SRMR = 0.005), and the model accounted for 18.9% and 20.2% of the variance in anxiety and depression symptoms, respectively.

### A and C Paths

As compared with the LL reference group, ML was associated with less minority stress (*a*_*1*_: *b* = −1.18, 95%BCa CI [−2.17, −0.15]) and fewer anxiety and depression symptoms (*c’*_*1*_: *b* = −0.72, 95%BCa CI [−1.34, −0.15]; *c’*_*4*_: *b* = −0.40, 95%BCa CI [−0.74, −0.08]). MH was associated with lower minority stress (*a*_*2*_: *b* =−1.47, 95%BCa CI [−2.63, −0.35]) but was not directly associated with each mental health outcome. HH was associated with lower minority stress (*a*_*3*_: *b* = −2.57, 95%BCa CI [−3.47, −1.68]) and fewer anxiety and depression symptoms (*c’*_*3*_: *b* = −0.90, 95%BCa CI [−1.46, −0.35]; *c’*_*6*_: *b* = −0.34, 95%BCa CI [−0.64, −0.03], respectively).

### B Paths

Each minority stressor reported by SMA was significantly associated with greater anxiety symptoms (*b*_*1*_: 95%BCa CI 0.23, 0.28) and depression symptoms (*b*_*2*_: 95%BCa CI 0.13, 0.16).

### Indirect and Total Effects

The indirect (*a*_*1*_*b*_*1*_: *b* = −0.30, 95%BCa CI [−0.56, −0.04]) and total (*c*_*1*_: *b* = −1.02, 95%BCa CI [−1.68, −0.40]) effects of ML on anxious symptoms through minority stress were statistically significant, as were the indirect symptoms (*a*_*1*_*b*_*2*_: *b* = −0.17, 95%BCa [CI −0.32, −0.02]) and total (*c*_*4*_: *b* = −0.57, 95%BCa CI [−0.94, −0.22]) effects of ML on depression symptoms. Overall, minority stress indirectly linked associations between ML and both anxiety symptoms and depression symptoms.

The indirect effects of MH on both anxiety symptoms (*a*_*2*_*b*_*1*_: *b* = −0.38, 95%BCa CI [−0.68, −0.09]) and depression symptoms (*a*_*2*_*b*_*2*_: *b* = −0.21, 95%BCa CI [−0.39, −0.05]) were significant. The total effects of MH on anxiety symptoms and depression symptoms were non-significant. Results indicated that minority stress was a mechanism linking associations between MH and anxiety symptoms and depression symptoms.

The indirect effect (*a*_*3*_*b*_*1*_: *b* = −0.66, 95%BCa CI [−0.91, −0.43]) and total effect (*c*_*3*_: *b* = −1.56, 95%BCa CI [−2.15, −0.97]) of HH on anxiety symptoms and the indirect effect (*a*_*3*_*b*_*2*_: *b* = −0.37, 95%BCa CI [−0.51, −0.24] and total effect (*c*_*6*_: *b* = −0.71, 95%BCa CI [−1.04, −0.39]) between HH and depression symptoms through minority stress were also significant. The effects of HH on both anxiety and depression symptoms were indirectly linked through minority stress.

**Table 2:** Mediation results

Direct Effects of Predictors on Outcomes	Path	b	SE	95% LCI	95% UCI
**Recent Minority Stress**					
*County-State Stigma (Ref: Low-Low)*					
Med-Low	*a*_1_	**−1.181**	**0.514**	**−2.173**	**−0.147**
Med-High	*a*_2_	**−1.468**	**0.586**	**−2.626**	**−0.346**
High-High	*a*_3_	**−2.572**	**0.458**	**−3.473**	**−1.675**
*Sex Designated at Birth (Ref: Female)*		0.314	0.407	−0.476	1.108
*Sexual Orientation (Ref: Bi/Pan/Queer)*		0.188	0.385	−0.566	0.926
*Race/Ethnicity (Ref: People of Color)*		**−1.154**	**0.383**	**−1.902**	**−0.395**
*Free/Reduced Price Lunch (Ref: Ineligible)*		**1.073**	**0.385**	**0.332**	**1.823**
**Anxious Symptoms**					
*County-State Stigma (Ref: Low-Low)*					
Med-Low	*c*’_1_	**−0.722**	**0.302**	**−1.335**	**−0.148**
Med-High	*c*’_2_	0.033	0.352	−0.652	0.723
High-High	*c*’_3_	**−0.900**	**0.284**	**−1.461**	**−0.350**
Recent Minority Stress	*b*_1_	**0.255**	**0.012**	**0.231**	**0.280**
*Sex Designated at Birth (Ref: Female)*		**−2.260**	**0.248**	**−2.751**	**−1.787**
*Sexual Orientation (Ref: Bi/Pan/Queer)*		−0.192	0.236	−0.662	0.266
*Race/Ethnicity (Ref: People of Color)*		**0.615**	**0.194**	**0.232**	**0.992**
*Free/Reduced Price Lunch (Ref: Ineligible)*		**0.579**	**0.223**	**0.130**	**1.004**
**Depressive Symptoms**					
*County-State Stigma (Ref: Low-Low)*					
Med-Low	*c*’_4_	**−0.403**	**0.167**	**−0.744**	**−0.079**
Med-High	*c*’_5_	0.122	0.208	−0.284	0.527
High-High	*c*’_6_	**−0.336**	**0.157**	**−0.645**	**−0.030**
Recent Minority Stress	*b*_2_	**0.145**	**0.007**	**0.130**	**0.159**
*Sex Designated at Birth (Ref: Female)*		**−1.652**	**0.138**	**−1.930**	**−1.382**
*Sexual Orientation (Ref: Bi/Pan/Queer)*		0.040	0.130	−0.220	0.290
*Free/Reduced Price Lunch (Ref: Ineligible)*		**0.502**	**0.127**	**0.256**	**0.752**
**Indirect and Total Effects on Anxiety Symptoms**	**Path**	***b***	***SE***	**95% LCI**	**95% UCI**
Med-Low					
Indirect	*a*_1_ *b*_1_	**−0.302**	**0.132**	**−0.560**	**−0.039**
Total effect, (*c’*_1_ + *a*_1_*b*_1_)	*c*_1_	**−1.023**	**0.327**	**−1.680**	**−0.395**
Med-High					
Indirect	*a*_2_ *b*_1_	**−0.375**	**0.151**	**−0.682**	**−0.092**
Total effect, (*c*’_2_ + *a*_2_*b*_1_)	*c*_2_	−0.342	0.374	−1.074	0.390
High-High					
Indirect	*a*_3_ *b*_1_	**−0.657**	**0.122**	**−0.906**	**−0.426**
Total effect, (*c*’_3_ + *a*_3_*b*_1_)	*c*_3_	**−1.557**	**0.298**	**−2.145**	**−0.968**
**Indirect and Total Effects on Depression Symptoms**	**Path**	***b***	***SE***	**95% LCI**	**95% UCI**
Med-Low					
Indirect	*a*_1_ *b*_2_	**−0.171**	**0.075**	**−0.316**	**−0.021**
Total effect, (*c’*_4_ + *a*_1_*b*_2_)	*c*_4_	**−0.573**	**0.182**	**−0.940**	**−0.217**
Med-High					
Indirect	*a*_2_ *b*_2_	**−0.212**	**0.086**	**−0.385**	**−0.052**
Total effect, (*c’*_5_ + *a*_2_*b*_2_)	*c*_5_	−0.091	0.221	−0.526	0.340
High-High					
Indirect	*a*_3_ *b*_2_	**−0.372**	**0.069**	**−0.513**	**−0.240**
Total effect, (*c’*_6_ + *a*_3_*b*_2_)	*c*_6_	**−0.708**	**0.167**	**−1.039**	**−0.387**

*Notes. N* = 2,558; *b*: unstandardized regression estimates; *SE* = standard errors; 95% LCI and UCI: lower and upper confidence intervals; all CIs are bias-corrected accelerated with 10,000 bootstrap samples. Significant CIs are presented in bold. Fit indices: χ^2^ = 3.864(1), *p* =.049, RMSEA = 0.033, SRMR = 0.005, CFI = 0.999, TLI = 0.967, NFI = 0.999

**Table 3:** Mediation model covariances

Variable	***b***	***SE***	**95% LCI**	**95% UCI**
Med-Low				
Med-High	**−0.031**	**0.002**	**−0.034**	**−0.027**
High-High	**−0.090**	**0.003**	**−0.096**	**−0.085**
Sex Designated at Birth	−0.002	0.004	−0.010	0.006
Sexual Orientation	−0.003	0.004	−0.012	0.005
Race/Ethnicity	**−0.021**	**0.004**	**−0.030**	**−0.013**
Free/Reduced Price Lunch	0.000	0.004	−0.008	0.009
Med-High				
High-High	**−0.043**	**0.002**	**−0.048**	**−0.039**
Sex Designated at Birth	0.004	0.003	−0.002	0.010
Sexual Orientation	0.004	0.003	−0.002	0.010
Race/Ethnicity	**0.014**	**0.003**	**0.008**	**0.020**
Free/Reduced Price Lunch	−0.001	0.003	−0.007	0.005
High-High				
Sex Designated at Birth	−0.001	0.005	−0.010	0.008
Sexual Orientation	−0.002	0.005	−0.011	0.007
Race/Ethnicity	**−0.029**	**0.005**	**−0.038**	**−0.019**
Free/Reduced Price Lunch	**−0.017**	**0.005**	**−0.026**	**−0.008**
Sex Designated at Birth				
Sexual Orientation	**0.096**	**0.004**	**0.087**	**0.105**
Race/Ethnicity	−0.009	0.005	−0.018	0.000
Free/Reduced Price Lunch	−0.003	0.005	−0.012	0.006
Sexual Orientation				
Race/Ethnicity	−0.003	0.005	−0.012	0.007
Free/Reduced Price Lunch	−0.003	0.005	−0.012	0.006
Race/Ethnicity				
Free/Reduced Price Lunch	**−0.052**	**0.005**	**−0.061**	**−0.043**
Anxiety Symptoms				
Depression Symptoms	**8.708**	**0.344**	**8.060**	**9.416**

*Notes. b*: unstandardized regression estimates; *SE* = standard errors; 95% LCI and UCI: lower and upper confidence intervals. Significant CIs are presented in bold

Among the covariates included in the path analysis, significant differences emerged by sex designated at birth, race/ethnicity, and lunch eligibility. Where people of color had significantly higher minority stress than White respondents, White SMA respondents had significantly higher anxiety symptoms than respondents of color. SMA designated female at birth had elevated anxiety and depression symptoms compared to SMA designated male at birth. SMA eligible for free or reduced-price lunch had elevated minority stress, anxiety symptoms, and depression symptoms compared to those who were free/reduced-price lunch ineligible. 

## Discussion

Study findings provide empirical support to theories that structural stigma is associated with health through psychosocial processes, such as exposure to minority stress. The study addressed a noted limitation in the structural stigma literature by examining differences between adolescents in differentially supportive counties in supportive and unsupportive states. Prior work has often compared supportive and unsupportive states without accounting for within-state heterogeneity, such as supportive oases in otherwise unsupportive states. Findings that adolescents benefitted most from combined county and state support, in terms of reporting lower minority stress, when compared with adolescents in unsupportive counties of unsupportive states provides support for the study hypothesis.

Results from this study also support the second hypothesis that minority stress indirectly links structural stigma and psychological distress (Hatzenbuehler, 2009, 2018; Meyer, 2003; Meyer et al., 2008). Study findings imply that both county and state structural stigma are associated with minority stress as well as greater anxiety and depression symptoms. State and especially local policymakers – and voters – must consider how LGBTQ-related policies may prevent or exacerbate SMA exposure to minority stress, and in turn increase health burden in this vulnerable population. For local policymakers in unsupportive states, findings point to the significant and meaningful impact their policymaking has in shaping county climate and either protecting or degrading SMA mental health.

Different findings for adolescents in relatively supportive counties of unsupportive states are particularly notable. Adolescents in relatively supportive counties of unsupportive states appear to benefit from “supportive oases” (e.g., supportive urban settings) in broadly unsupportive state environments, as evidenced by significantly lower minority stress and mental health symptoms and significant direct and indirect associations in path analysis. Compared to adolescents in unsupportive counties of unsupportive states, adolescents in relatively unsupportive counties of otherwise supportive states had significantly lower minority stress, which indirectly linked to fewer anxiety and depression symptoms.

Relatively fewer county protections in generally supportive states may nonetheless be accompanied by unmeasured, heightened local antagonism. Although the state score counted both pro- and anti-LGBTQ policies, the county structural stigma variable only counted the presence or absence of protections, not the presence or absence of antagonistic policies. It may be that antagonistic county policies and institutions (e.g., highly active local anti-LGBTQ institutions) not measured by this study could further account for variation in the outcomes, including for adolescents in relatively unsupportive counties of otherwise supportive states. Further research is needed to explore the contributions of other variables at structural and individual levels, such as social service access, stress unrelated to sexual minority status, political engagement, and religiosity.

This study also identified significant differences in minority stress exposure and mental health problems by demographic group. Findings of greater minority stress, anxiety, and depression symptoms for free/reduced-price lunch eligible than ineligible SMA reflect similar findings in the literature Parra et al., 2023, which found that SMA family rejection indirectly linked familial job loss at COVID-19 pandemic onset with psychological distress symptoms within a sub-sample of this study at another wave of data collection. Similar research on intersectional minority stress disparities with sexual minority adults has identified that Black and Latinx sexual minority adults report more minority stress than White sexual minority adults, but that these effects also interact significantly with socioeconomic status (Shangani et al., 2020). In this study, SMA of color reported significantly more minority stress than White SMA, whereas White SMA exhibited significantly greater anxiety symptoms compared to SMA of color. Further, this pattern of findings may reflect the resilience hypothesis, that people of color experience more stressors but cope more efficiently with those stressors, such that they exhibit lower psychological distress (Meyer, 2010). However, other studies have presented contradictory findings that SMA of color do not experience more stress and have pointed to the risk of furthering stereotypes of heterosexism within communities of color (Moradi et al., 2010). Further research needs to explore, with larger samples of SMA of color, these patterns and their associations with structural stigma using intersectionality perspectives. Findings of heightened mental health symptoms among SMA designated female compared with those designated male reflect research with heterosexual samples, but sexual and gender minority research indicates different depression and anxiety symptom patterns by gender and sexual orientation (Smalley et al., 2016).

Findings need to be read considering study limitations. The cross-sectional design precludes causative conclusions or a directional pattern of effects over time. There was a threat of misclassifying adolescents into counties by zip code. Of the full sample, 742 adolescents reported residence in zip codes that crossed multiple counties, but most of those zip codes crossed counties with the same county scores such that their misclassification was numerically inconsequential. The remaining 170 adolescents (6.6% of the sample) could have lived in one or another county with different county stigma scores. A sensitivity analysis excluding these 170 cases yielded the same pattern of results (data not shown). The decision to combine bisexual, pansexual, queer, and “another” sexual orientation groups and groups of people of color for parsimony in path analysis inhibited investigation of differences within these groups.

Though social media recruitment advertising was stratified by gender, geography, and urbanicity, the use of online surveys excluded SMA who lack internet access, and some groups remain underrepresented in the sample, such as people designated male at birth. Additionally, the parent study excluded transgender participants at baseline, so findings may not generalize to gender minority adolescents. Two county policies (conversion therapy bans and non-discrimination ordinances) were coded “yes” if the state had adopted those policies, which could have contributed to multicollinearity; collapsing these variables into a single county-state variable sought to address this issue.

## Conclusion

Findings indicate that county and state protections are associated with minority stress and mental health symptoms in SMA. County and state protections were associated with lower exposure to minority stress, which in turn was associated with both fewer anxiety and depression symptoms. This study shows that both county and state protections support SMA mental health and findings point, especially, to the need to adopt local protections to support SMA mental health, ensure that state-level protections benefit all adolescents, and consider county variation when measuring the effects of state-level structural stigma in research. These findings also suggest that policies designed to discriminate directly or indirectly based on sexual orientation may have negative costs to society that should be factored into policy impact analyses. Further research may investigate structural and individual-level moderators of these associations, causal and longitudinal relationships between county and state structural stigma on minority stress and health, and how changes in county and state structural stigma contribute to change in SMA wellbeing.

## Data Availability

The datasets generated during and/or analyzed during the current study are available from the corresponding author on reasonable request.

## References

[CR1] Baams, L., Dubas, J. S., Russell, S. T., Buikema, R. L., & van Aken, M. A. (2018). Minority stress, perceived burdensomeness, and depressive symptoms among sexual minority youth. *Journal of Adolescence,**66*, 9–18. 29723686 10.1016/j.adolescence.2018.03.015 PMC6919272

[CR3] Chien, Y. S., Schwartz, G., Huang, L., & Kawachi, I. (2022). State LGBTQ policies and binge drinking among sexual minority youth in the US: A multilevel analysis. *Social Psychiatry and Psychiatric Epidemiology*, *57*(1), 183–194. 34143248 10.1007/s00127-021-02119-4

[CR4] Cramer, R., Hexem, S., LaPollo, A., Cuffe, K. M., Chesson, H. W., & Leichliter, J. S. (2017). State and local policies related to sexual orientation in the United States. *Journal of Public Health Policy,**38*(1), 58–79. 28275249 10.1057/s41271-016-0037-9 PMC6752226

[CR5] Cyrus, K. (2017). Multiple minorities as multiply marginalized: Applying the minority stress theory to LGBTQ people of color. *Journal of Gay & Lesbian Mental Health*, *21*(3), 194–202.

[CR6] D’Augelli, A. R. (2002). Mental health problems among lesbian, gay, and bisexual youths ages 14 to 21. *Clinical Child Psychology and Psychiatry*, *7*(3), 433–456.

[CR7] Dahl, R. E., & Gunnar, M. R. (2009). Heightened stress responsiveness and emotional reactivity during pubertal maturation: Implications for psychopathology. *Development and Psychopathology*, *21*(1), 1–6. 19144219 10.1017/S0954579409000017

[CR8] De Girolamo, G., Dagani, J., Purcell, R., Cocchi, A., & McGorry, P. (2012). Age of onset of mental disorders and use of mental health services: Needs, opportunities and obstacles. *Epidemiology and Psychiatric Sciences,**21*(1), 47–57. 22670412 10.1017/s2045796011000746

[CR9] Domina, T., Pharris-Ciurej, N., Penner, A. M., Penner, E. K., Brummet, Q., Porter, S. R., & Sanabria, T. (2018). Is free and reduced-price lunch a valid measure of educational disadvantage? *Educational Researcher*, *47*(9), 539–555.

[CR10] Duncan, D. T., Hatzenbuehler, M. L., & Johnson, R. M. (2014). Neighborhood-level LGBT hate crimes and current illicit drug use among sexual minority youth. *Drug and Alcohol Dependence*, *135*, 65–70. 24326203 10.1016/j.drugalcdep.2013.11.001 PMC3919662

[CR11] Fulginiti, A., Goldbach, J. T., Mamey, M. R., Rusow, J., Srivastava, A., Rhoades, H., & Marshal, M. P. (2020). Integrating minority stress theory and the interpersonal theory of suicide among sexual minority youth who engage crisis services. *Suicide and Life-Threatening Behavior*, *50*(3), 601–616. 32048340 10.1111/sltb.12623

[CR52] Goldbach, J. T., Tanner-Smith, E. E., Bagwell, M., & Dunlap, S. (2014). Minority stress and substance use in sexual minority adolescents: *A meta-analysis*. Prevention Science, 15(*3*), 350–363. 10.1007/S11121-013-0393-7 10.1007/s11121-013-0393-7 23605479

[CR51] Goldbach, J. T., Schrager, S. M., Mamey, M. R., & Rhoades, H. (2021). Confirming the reliability and validity of the Sexual Minority Adolescent Stress Inventory in a national sample of sexual minority adolescents. *Frontiers in psychology*, 12, 720199. 10.3389/fpsyg.2021.720199 10.3389/fpsyg.2021.720199 PMC8438190 34531800

[CR12] Gordon, J. H., Tran, K. T., Visoki, E., Argabright, S. T., DiDomenico, G. E., Saiegh, E., & Barzilay, R. (2024). The role of individual discrimination and structural stigma in the mental health of sexual minority youth. *Journal of the American Academy of Child and Adolescent Psychiatry,**63*(2), 231–244. 37422106 10.1016/j.jaac.2023.05.033 PMC10770287

[CR13] Guardian, T. (2016). Election 2016 Results. https://www.theguardian.com/us-news/ng-interactive/2016/nov/08/us-election-2016-results-live-clinton-trump?view=boardtype=presidential

[CR14] Hall, W. J. (2018). Psychosocial risk and protective factors for depression among lesbian, gay, bisexual, and Queer youth: A systematic review. *Journal of Homosexuality*, *65*(3), 263–316. 28394718 10.1080/00918369.2017.1317467 PMC5634914

[CR15] Hall, W. J., Dawes, H. C., & Plocek, N. (2021). Sexual orientation identity development milestones among lesbian, gay, bisexual, and Queer people: A systematic review and meta-analysis. *Frontiers in Psychology*, *12*, 753954. 34777153 10.3389/fpsyg.2021.753954 PMC8581765

[CR16] Harris, K. C., Whitsel, E., & Hussey, J. (2009). The National longitudinal study of adolescent to adult health: research design. In. http://www.cpc.unc.edu/projects/addhealth/design 10.1093/ije/dyz115 PMC6857761 31257425

[CR17] Hatzenbuehler, M. L. (2009). How does sexual minority stigma “get under the skin”? A psychological mediation framework. *Psychological Bulletin,**135*(5), 707. 19702379 10.1037/a0016441 PMC2789474

[CR18] Hatzenbuehler, M. L. (2018). Structural stigma and health. In J. F. D. Brenda, Major, & B. G. Link (Eds.), *The Oxford handbook of Stigma, Discrimination, and health* (pp. 105–124). Oxford University Press.

[CR19] Hatzenbuehler, M. L., & Keyes, K. M. (2013). Inclusive anti-bullying policies and reduced risk of suicide attempts in lesbian and gay youth. *Journal of Adolescent Health*, *53*(1), S21–S26. 10.1016/j.jadohealth.2012.08.010 PMC3696185 23790196

[CR20] Hatzenbuehler, M. L., & Link, B. G. (2014). Introduction to the special issue on structural stigma and health. *Social Science & Medicine*. 10.1016/j.socscimed.2013.12.017 10.1016/j.socscimed.2013.12.017 24445152

[CR21] Homan, P. (2019). Structural sexism and health in the United States: A new perspective on health inequality and the gender system. *American Sociological Review,**84*(3), 486–516.

[CR22] Kann, L., McManus, T., Harris, W. A., Shanklin, S. L., Flint, K. H., Queen, B., & Thornton, J. (2018). Youth risk behavior surveillance—United States, 2017. *MMWR Surveillance Summaries*, *67*(8), 1. 10.15585/mmwr.ss6708a1 PMC6002027 29902162

[CR23] Kessler, R. C., Angermeyer, M., Anthony, J. C., De Graaf, R., Demyttenaere, K., Gasquet, I., & Haro, J. M. (2007). Lifetime prevalence and age-of-onset distributions of mental disorders in the World Health Organization’s World Mental Health Survey Initiative. *World Psychiatry,**6*(3), 168. 18188442 PMC2174588

[CR24] Local Nondiscrimination Ordinances Movement Advancement Project. https://www.lgbtmap.org/equality-maps/non_discrimination_ordinances

[CR25] MacKinnon, D. (2012). *Introduction to statistical mediation analysis*. Routledge.

[CR26] Melchior, L. A., Huba, G., Brown, V. B., & Reback, C. J. (1993). A short depression index for women. *Educational and Psychological Measurement,**53*(4), 1117–1125.

[CR27] Meyer, I. H. (2003). Prejudice, social stress, and mental health in lesbian, gay, and bisexual populations: Conceptual issues and research evidence. *Psychological Bulletin*, *129*(5), 674. 12956539 10.1037/0033-2909.129.5.674 PMC2072932

[CR28] Meyer, I. H. (2010). Identity, stress, and resilience in lesbians, gay men, and bisexuals of color. *The Counseling Psychologist*, *38*(3), 442–454. 10.1177/0011000009351601 PMC3860594 24347674

[CR29] Meyer, I. H., Schwartz, S., & Frost, D. M. (2008). Social patterning of stress and coping: Does disadvantaged social statuses confer more stress and fewer coping resources? *Social Science & Medicine,**67*, 368–397. 18433961 10.1016/j.socscimed.2008.03.012 PMC2583128

[CR30] Montoya, A. K., & Hayes, A. F. (2017). Two-condition within-participant statistical mediation analysis: A path-analytic framework. *Psychological Methods*, *22*(1), 6. 27362267 10.1037/met0000086

[CR31] Moradi, B., Wiseman, M. C., DeBlaere, C., Goodman, M. B., Sarkees, A., Brewster, M. E., & Huang, Y. P. (2010). LGB of color and white individuals’ perceptions of heterosexist stigma, internalized homophobia, and outness: Comparisons of levels and links. *The Counseling Psychologist*, *38*(3), 397–424.

[CR49] O’Brien, R. P., Rhoades, H., Cabrera Jr, J. R., Parra, L. A., Rusow, J. A., Schrager, S. M., & Goldbach, J. T. (2023). Associations Between State Legislative Activity, Minority Stress, and Suicide Attempt Among Sexual Minority Adolescents. *Annals of LGBTQ Public and Population Health*. 10.1891/LGBTQ-2022-0022

[CR32] Pachankis, J. E., Hatzenbuehler, M. L., & Starks, T. J. (2014). The influence of structural stigma and rejection sensitivity on young sexual minority men’s daily tobacco and alcohol use. *Social Science & Medicine,**103*, 67–75. 24507912 10.1016/j.socscimed.2013.10.005 PMC5793849

[CR33] Patterson, C. J. (2008). Sexual orientation across the life span: Introduction to the special section. *Developmental Psychology*, *44*(1), 1. 18193999 10.1037/0012-1649.44.1.1

[CR48] Parra, L. A., Bell, T. S., Benibgui, M., Helm, J. L., & Hastings, P. D. (2018). The buffering effect of peer support on the links between family rejection and psychosocial adjustment in LGB emerging adults. *Journal of Social and Personal Relationships*, 35(*6*), 854–871. 10.1177/0265407517699713

[CR53] Parra, L. A., O’Brien, R. P., Schrager, S. M., & Goldbach, J. T. (2023). COVID-19-related household job loss and mental health in a nationwide United States sample of sexual minority adolescents. *Behavioral medicine*, 49(*1*), 62–71. 10.1080/08964289.2021.1977604 10.1080/08964289.2021.1977604 PMC11453117 34749595

[CR34] Perez-Brumer, A., Day, J. K., Russell, S. T., & Hatzenbuehler, M. L. (2017). Prevalence and correlates of suicidal ideation among transgender youth in California: Findings from a representative, population-based sample of high school students. *Journal of the American Academy of Child and Adolescent Psychiatry,**56*(9), 739–746. 28838578 10.1016/j.jaac.2017.06.010 PMC5695881

[CR35] Preacher, K. J., & Hayes, A. F. (2008). *Assessing mediation in communication research*. The Sage sourcebook of advanced data analysis methods for communication ….

[CR36] Rabasco, A., & Andover, M. (2020). The influence of state policies on the relationship between minority stressors and suicide attempts among transgender and gender-diverse adults. *LGBT Health,**7*(8), 457–460. 33090075 10.1089/lgbt.2020.0114

[CR37] Raifman, J., Moscoe, E., Austin, S. B., & McConnell, M. (2017). Difference-in-differences analysis of the association between state same-sex marriage policies and adolescent suicide attempts. *JAMA Pediatrics*, *171*(4), 350–356. 28241285 10.1001/jamapediatrics.2016.4529 PMC5848493

[CR38] Rosseel, Y. (2012). Lavaan: An R package for structural equation modeling and more. Version 0.5–12 (BETA). *Journal of Statistical Software,**48*(2), 1–36.

[CR39] Russell, S. T., & Fish, J. N. (2016). Mental health in lesbian, gay, bisexual, and transgender (LGBT) youth. *Annual Review of Clinical Psychology*, *12*(1), 465–487. 26772206 10.1146/annurev-clinpsy-021815-093153 PMC4887282

[CR40] Russell, S. T., & Fish, J. N. (2019). Sexual minority youth, social change, and health: A developmental collision. *Research in Human Development*, *16*(1), 5–20. 31602178 10.1080/15427609.2018.1537772 PMC6786797

[CR41] Saewyc, E. M., Li, G., Gower, A. L., Watson, R. J., Erickson, D., Corliss, H. L., & Eisenberg, M. E. (2020). The link between LGBTQ-supportive communities, progressive political climate, and suicidality among sexual minority adolescents in Canada. *Preventive Medicine*, *139*, 106191. 32653353 10.1016/j.ypmed.2020.106191 PMC8474062

[CR50] Schrager, S. M., Mamey, M. R., Rhoades, H., & Goldbach, J. T. (2022). Adolescent stress experiences over time study (ASETS) protocol: design and methods of a prospective longitudinal study of sexual minority adolescents in the USA. *BMJ open*, 12(*3*), e054792. 10.1136/bmjopen-2021-054792 10.1136/bmjopen-2021-054792 PMC8915334 35264352

[CR42] Shangani, S., Gamarel, K. E., Ogunbajo, A., Cai, J., & Operario, D. (2020). Intersectional minority stress disparities among sexual minority adults in the USA: The role of race/ethnicity and socioeconomic status. *Culture, Health & Sexuality,**22*(4), 398–412. 10.1080/13691058.2019.1604994 PMC6884660 31144598

[CR43] Smalley, K. B., Warren, J. C., & Barefoot, K. N. (2016). Variations in psychological distress between gender and sexual minority groups. *Journal of Gay & Lesbian Mental Health*, *20*(2), 99–115.

[CR44] Spitzer, R. L., Kroenke, K., Williams, J. B., & Löwe, B. (2006). A brief measure for assessing generalized anxiety disorder: The GAD-7. *Archives of Internal Medicine,**166*(10), 1092–1097. 16717171 10.1001/archinte.166.10.1092

[CR45] Warbelow, S., Oakley, C., & Kutney, C. (2019). *State equality index 2019*. In: Human Rights Campaign Foundation.

[CR46] Williams, T., Connolly, J., Pepler, D., & Craig, W. (2005). Peer victimization, social support, and psychosocial adjustment of sexual minority adolescents. *Journal of Youth and Adolescence*, *34*(5), 471–482.

[CR2] WWAMI Rural Health Research Center. (2022). Using RUCA Data. Accessed at: https://depts.washington.edu/uwruca/ruca-uses.php

[CR47] Zhao, X., LynchJr, J. G., & Chen, Q. (2010). Reconsidering Baron and kenny: Myths and truths about mediation analysis. *Journal of Consumer Research*, *37*(2), 197–206.

